# *Porphyromonas gingivalis*: A key role in Parkinson's disease with cognitive impairment?

**DOI:** 10.3389/fneur.2022.945523

**Published:** 2022-07-26

**Authors:** Dongcheng Li, Tengzhu Ren, Hao Li, Geng Liao, Xiong Zhang

**Affiliations:** ^1^Department of Neurology, Affiliated Maoming People's Hospital, Southern Medical University, Maoming, China; ^2^Department of Neurology, Guangdong Second Provincial General Hospital, Guangzhou, China

**Keywords:** Parkinson's disease, cognitive impairment, *Porphyromonas gingivalis*, neuroinflammation, α-Synuclein, amyloid-β, gut microbiota

## Abstract

Cognitive impairment (CI) is a common complication of Parkinson's disease (PD). The major features of Parkinson's disease with cognitive impairment (PD-CI) include convergence of α-Synuclein (α-Syn) and Alzheimer's disease (AD)-like pathologies, neuroinflammation, and dysbiosis of gut microbiota. *Porphyromonas gingivalis* (*P. gingivalis*) is an important pathogen in periodontitis. Recent research has suggested a role of P. gingivalis and its virulence factor in the pathogenesis of PD and AD, in particular concerning neuroinflammation and deposition of α-Synuclein (α-Syn) and amyloid-β (Aβ). Furthermore, in animal models, oral *P. gingivalis* could cause neurodegeneration through regulating the gut-brain axis, suggesting an oral-gut-brain axis might exist. In this article, we discussed the pathological characteristics of PD-CI and the role of *P. gingivalis* in them.

## Introduction

Parkinson's disease (PD) is a prevalent neurodegenerative disease caused by the death of dopaminergic neurons in the substantia nigra pars compacta (SNpc). Cognitive impairment (CI) is a common complication of PD non-motor symptoms, including PD with mild cognitive impairment (PD-MCI) and PD dementia (PDD). PD-MCI, as an independent risk factor for PDD (1), is characterized by the transitional state that fails to meet the diagnostic criteria for PDD. A 5-year follow-up study of patients with PD showed that the cumulative incidence of PD-MCI in patients aged ≥ 65 years is 41.3% after 5 years, and the conversion rate of patients with PD-MCI progressing to PDD within 5 years is about 39–50% (2).

The pathogenesis of PD-CI is unclear. Current studies have found that PD-CI-related pathologies include the deposition of α-Synuclein (α-Syn), Alzheimer's disease-type pathologies (amyloid-β, tau, and neurofibrillary tangles), and neuroinflammation (3–5). Recently, many studies have shown a correlation between *Porphyromonas gingivalis* (*P. gingivalis*) infection and PD (5–8). Interestingly, *P. gingivalis* has recently been shown to be associated with cognitive impairment and a potential microbial driver in Alzheimer s disease (AD) (9–15). Therefore, this review summarizes the literature on the potential role of *P. gingivalis* in PD-CI development and is aimed as a reference for further research and therapy.

## Porphyromonas gingivalis

*Porphyromonas gingivalis* is a Gram-negative, anaerobic, and rod-shaped bacteria that colonizes the oral epithelium and is an important component of subgingival microbiomes. *P. gingivalis* is responsible for the chronic form of periodontitis through its capacity to remodel the commensal bacterial community, which promotes a state of dysbiosis (16). It can engineer its environment or modify the host's immune response to modulate the entire ecosystem. It can also persist in host tissues through unique and intricate mechanisms, such as the alteration of inflammatory signaling pathways, the complement system, the cell cycle, apoptosis, and the interaction with various host receptors (17). During common activities, such as brushing, flossing, chewing, and dental procedures (18), *P. gingivalis* invades the vasculature from the infected periodontal pocket. Furthermore, a study using an animal model showed that it can also increase the permeability of the blood-brain barrier (BBB) and facilitate access of bacteria into the brain (19).

The strategies and pathogenicity of *P. gingivalis* largely rely on its various virulence factors. Among these, the secretory components and gingipains are major virulence factors, consist of lysine-gingipain (Kgp) and arginine-gingipain (Rgp), which play essential roles in host colonization, host defense deactivation, tissue destruction, and nutrient acquisition (20). In addition, lipopolysaccharide (LPS), a structural component, is also a crucial pathogenic factor that can trigger the innate immune response *via* the activation of toll-like receptors (TLRs), leading to more lasting destruction of periodontal tissues (21). *P. gingivalis* LPS induces the expression of interleukin (IL)-6 and C-C motif chemokine ligand 2 (CCL2) in the brain microvascular endothelial cells, which may contribute to dysfunction of the BBB and subsequent neurological disorders (22, 23). Furthermore, *P. gingivalis* LPS can induce IL-8 elevation (24) and IL-10 decline (25) in the brain, which is consistent with cytokine changes in the cerebrospinal fluid (CSF) of patients with PD (26). Indeed, gingipains and LPS have attracted growing attention as virulence factors of *P. gingivalis* and recent studies have found that these two factors are present in the blood circulation and brain of PD and AD populations (5, 27). In addition to neurodegenerative diseases, studies have shown that *P. gingivalis* may be associated with other systemic inflammatory diseases, such as type 2 diabetes mellitus, rheumatoid arthritis, and cardiopulmonary disease (28).

## Pathological characteristics of PD-CI

### α-Syn and AD-type pathologies

Current neuropathological studies indicated that the convergence of α-Syn and AD-type pathologies is the major pathological features of PD-CI (4).

The misfolding and aggregation of aberrant α-Syn in the patients' brain are the major characteristics of PD (29) that result in neuron loss and the clinical syndrome of idiopathic PD (30). Moreover, it was found that pathogenic α-Syn can transfer between cells leading to neurodegeneration (30–32). The importance of α-Syn is further emphasized in PD-CI studies which showed that α-Syn pathology is more extensive and severe in PDD than in PD without dementia. PDD cases are almost exclusively of the predominant stage of α-Syn pathology in the limbic system or neocortex and α-Syn pathology level can distinguish between PD and PDD (3, 33, 34). The progression of α-Syn pathology stage or cortical α-Syn pathology burden is highly correlated with the cognitive level decline (35–37).

However, despite α-Syn pathology being the major driving force of the development in cognitive impairment in patients with PD, AD pathology was also found to play an important role in PD-CI (4). Many clinical studies revealed that the tau concentration in the CSF is associated with cognitive impairment in Parkinson's disease (38–41). In some studies, AD neuropathology seemed to be even more associated with PDD than with α-Syn pathology, but most of these patients with PD were assigned a diagnosis of PDD + AD (3, 4, 33). In fact, α-Syn pathological levels in the cortex and limbic system of patients with PDD + AD appeared to be higher than those of patients with PDD without AD comorbidities. An increased severity of cortical senile plaques (SPs) and burden of neurofibrillary tangles (NFTs) were also related to an increased cortical α-Syn pathological density (3, 33, 36, 42). Recently, Bassi et al. demonstrated that amyloid-β (Aβ) deposits dramatically accelerate α-Syn pathogenesis and spread throughout the brain after injecting α-Syn preformed fibrils into mice with abundant Aβ plaques. Recent pathological studies *in vitro* showed that AD-related pathologies could exacerbates α-Syn seeding activity and neurotoxicity (43, 44), suggesting an interaction between α-Syn pathology and AD-type pathologies in PD-CI.

### Neuroinflammation

In addition to α-Syn and AD pathology, neuroinflammation is also a crucial factor in PD-CI. Although it is not clear how inflammation contributes to the pathogenesis of PD-CI, it is universally acknowledged that both central and peripheral inflammations contribute to the progression of neurodegeneration in PD-CI. A study by Lindqvist et al. (45) indicated that PD non-motor features were associated with higher CSF levels of inflammatory markers. The C-reactive protein (CRP) level in the CSF of patients with PDD was significantly higher than that of patients with non-demented PD (*p* = 0.032) (45). In addition, a large cohort study of newly diagnosed patients with PD showed that higher levels of interferon gamma (IFN-γ), TNF-α, and CRP in blood are associated with a lower Mini-Mental State Examination (MMSE) score in patients with PD, and that IL-1β and IL-2 are related to a faster rate of cognitive decline (46). Recently, an animal study demonstrated that peripherally induced neuroinflammation potentiates the harmful effects of α-Syn. Furthermore, in genetic pathologic PD models, LPS-induced neuroinflammation aggravated cognitive deficits (47).

Neuroinflammation is mainly promoted by microglia and astrocytes. Under chronic peripheral inflammation, microglial and astroglial cells are overactivated by toll-like receptors (TLRs), resulting in the release of various inflammatory cytokines (e.g., TNF- a, IL-6, and CXCL1), leading to chronic neuroinflammation (48, 49). Neuroinflammation induces and exacerbates α-Syn and AD-type pathologies. Activated glial cells interact with α-Syn and AD-type pathologies (48, 50), and mediate their detrimental effects on both memory and neuroinflammation (47). These processes can promote a vicious circle and lead to PD-CI.

### Dysbiosis of gut microbiota

Increasing evidence from studies of the gut–brain axis has suggested that the gut microbiome plays a critical role in neurodegenerative diseases, such as AD and PD (51). Previously, our research (52) has found that the gut microbiota of patients with PD-MCI was significantly altered compared with those healthy controls (HCs) and patients with PD with normal cognition (PD-NC). This is particularly manifesting in enriched genera from Porphyromonadaceae family, providing powerful evidence that the dysbiosis of gut microbiota may contribute to PD-CI (52).

Although the role of intestinal flora alteration in PD-CI is not completely known, we can still deduce hints from the results of recent studies. First, according to Braak's hypothesis (53), the accumulation of aberrant α-Syn is initiated in the gut and propagates *via* the vagus nerve to the brain. Furthermore, growing evidence showed that α-Syn may be transmitted in a prion-like manner (54–57). Recent studies also supported Braak's hypothesis in the etiology of PD and the prion-like theory (58), and indicated that the prion-like seeding activity of aberrant α-Syn may be related with its post-translational modifications (such as, carboxy-truncation) or oligomerization (59, 60). Several factors, such as antibiotics, diet, birth mode, or stress may trigger or promote the translocation of intestinal microorganisms and microbial products (such as, LPS), which would cause oxidative stress and mucosal inflammation, and promote the accumulation of α-Syn in the enteric nervous system (ENS) (61–63). Indeed, recent experiments have shown that the LPS of intestinal microorganisms can regulate the aggregation and toxicity of α-Syn and lead to cognitive decline (47, 64). Second, LPS from gut microbiota can disrupt the integrity of the BBB (62, 65, 66), which may promote neuroinflammation and SNpc damage. Finally, an experiment using a mouse model showed that microbiome disturbances have influences on microglia-mediated brain Aβ deposition (67). Collectively, these results suggest that alterations in the intestinal flora may facilitate the deposition of α-Syn and Aβ, and neuroinflammation, resulting in the induction induce PD-CI.

## *P. gingivalis* and PD

In earlier studies, the view was that motor and cognitive disturbances that are caused by PD, could contribute to the progression of periodontal disease (68, 69). However, as periodontal disease is gradually found to be related to the onset and progression of AD (10), a reverse correlation between periodontal disease and PD is increasingly attracting attention (68, 70). The studies by Chen et al. (6, 71) showed that the risk of developing PD in patients with periodontitis is significantly higher than that in controls [adjusted hazard ratio (*HR*) = 1.431, *p* = 0.002] and patients without periodontitis who had a significantly lower risk of developing PD after dental scaling over 5 consecutive years [adjusted odds ratio (*OR*) = 0.204, *p* = 0.0399]. Another similar study by Jeong et al. (7) suggested a weak association between periodontitis and PD (log rank *p* < 0.001).

Recently, a study by Adams et al. (5) showed that gingipain R1 (RgpA), produced by *P. gingivalis*, is present in the blood circulation, highlighting the potential involvement of *P. gingivalis* in Parkinson's disease. In addition, Adams et al. found that the whole blood of patients with PD is hypercoagulable, due to the presence of hyperactivated platelets and fibrin(ogen) amyloid features. These results are consistent with previous finding (72) that clots are denser and hyperclottable in patients with PD. Moreover, preliminary data suggested a role of *P. gingivalis* LPS and gingipain in the systemic inflammatory and hypercoagulable pathology of PD.

The R1441G mutation in the leucine-rich repeat kinase 2 (LRRK2) gene results in late-onset PD (73). A recent animal study by Feng et al. found that (8), orally administrating live *P. gingivalis* to LRRK2 R1441G mice three times a week for 1 month, can induce a mutant LRRK2-dependent reduction of dopaminergic neurons in the substantia nigra, an increase in mutant LRRK2 expression, and the activation of microglia, leading to peripheral IL-17A secretion and IL-17 receptor A (IL-17RA) upregulation. These results provide further evidence on the correlation between *P. gingivalis* and PD.

## *P. gingivalis* and cognitive impairment

Alzheimer's disease is the most common neurodegenerative disorder that causes cognitive impairment (49). Many studies have revealed that periodontitis increases the risk of AD (9–11). *P. gingivalis*, the main pathogen in periodontitis, was shown to play an important role in AD. In a cross-sectional study, patients with high *P. gingivalis* IgG had worse delayed verbal memory and impaired subtraction in a dose-response relationship (12). The study by Stein et al. indicated that after adjusting for confounders, such as age and smoking, serum antibody levels of *P. gingivalis* were higher in AD patients with cognitive impairment and positively correlated with the stage of AD development (13). Furthermore, *P. gingivalis* DNA, LPS (14), and gingipains (15) have been recently detected in the brains of patients with AD.

Amyloid-β plaques, neurofibrillary tangles, and neuroinflammation are the major hallmarks of AD. *P. gingivalis* may promote the progression of AD by contributing to these pathologies. The study by Wu et al. (74) showed that chronic exposure to *P. gingivalis* LPS for 5 consecutive weeks causes AD-like phenotypes, such as learning and memory deficits, microglia-mediated neuroinflammation and Aβ accumulation in neurons of middle-aged wild-type mice. In addition, cathepsin B (CatB) may be crucial for this process, as *P. gingivalis* LPS-induced AD-like phenotypes were found to be CatB-dependent. Interestingly, CatB plays a critical role in peripheral Aβ generation, as *P. gingivalis* infection induces the production of Aβ in inflammatory macrophages *via* activating the CatB/NF-κB signaling (75). Subsequently, a study by Zeng et al. (76) indicated that *P. gingivalis* infection can promote the CatB/NF-κB-dependent receptor for advanced glycation end (RAGE) expression in cerebral endothelial cells, which mediates the influx of peripheral Aβ into the brain across the BBB. These results revealed a potential pathogenesis of AD, associated with *P. gingivalis* induction of induced peripheral Aβ production and influx, resulting in AD-type pathologies in the brain, and in which CatB plays an important role.

To the best of our knowledge, no study has directly demonstrated the correlation between *P. gingivalis* and PD-CI. However, in a report by La Vitola et al. (47), it was shown that *Escherichia coli* (*E. coli*) LPS-induced neuroinflammation can aggravate the toxic effects of α-Syn and cognitive deficits. However, in the study by Zhang et al., although either *P. gingivalis* LPS or *E. coli* LPS was shown to impair spatial learning and memory in the MWM test, no significant differences were observed between the effects of the two LPS species (24). These results suggest that, despite their structural differences, *P. gingivalis* LPS may have similar mechanisms to those of *E. coli* LPS in exacerbating the α -Syn detrimental effects and cognitive impairment.

## *P. gingivalis* and gut microbiota

According to previous reports, the density of *P. gingivalis* in saliva of patients with severe periodontitis can reach to 10^6^/ml (77–79). Since humans produce 1–1.5 L of saliva a day, about 10^12^-10^13^ *P. gingivalis* bacteria might be daily swallowed by patients with severe periodontitis (80). Animal studies showed that the oral administration of *P. gingivalis* at simulated doses causes gut microbiota dysbiosis and gut permeability impairment, resulting in systemic inflammation in mice (80, 81). It was reported that *P. gingivalis*-induced dysbiosis is related to arthritis (65, 82), type 2 diabetes (83), and non-alcoholic fatty liver disease (NAFLD) (84).

As gut microbiota plays an important role in the gut-brain axis, whether *P. gingivalis* promotes neurodegenerative diseases through regulating the gut-brain axis, is still under research. Feng et al. (8) have found that the oral administration of *P. gingivalis* can lead to gut permeability impairment and an increase in the accumulation of α-Syn in the colon neurons of LRRK2 R1441G mice. Although the authors did not detect α-Syn in the brain or small intestine, this finding supports the previous point that the accumulation of aberrant α-Syn is initiated in the gut (53, 58). In addition, emerging evidence by Chi et al. (85) shows that oral *P. gingivalis* induces gut microbiota dysbiosis, exacerbates neuroinflammation, and ultimately lead to a decline in cognitive function. Furthermore, the number of neurons in the hippocampal and cortical regions was significantly decreased, and amyloid plaques appeared in brain. The results from these two studies are consistent with the characteristics of PD-CI mentioned above, indicating an oral-gut-brain axis may exist and contribute to PD-CI, which is worthy of further confirmation in future studies.

## How is *P. gingivalis* involved in PD-CI?

**Figure 1:**
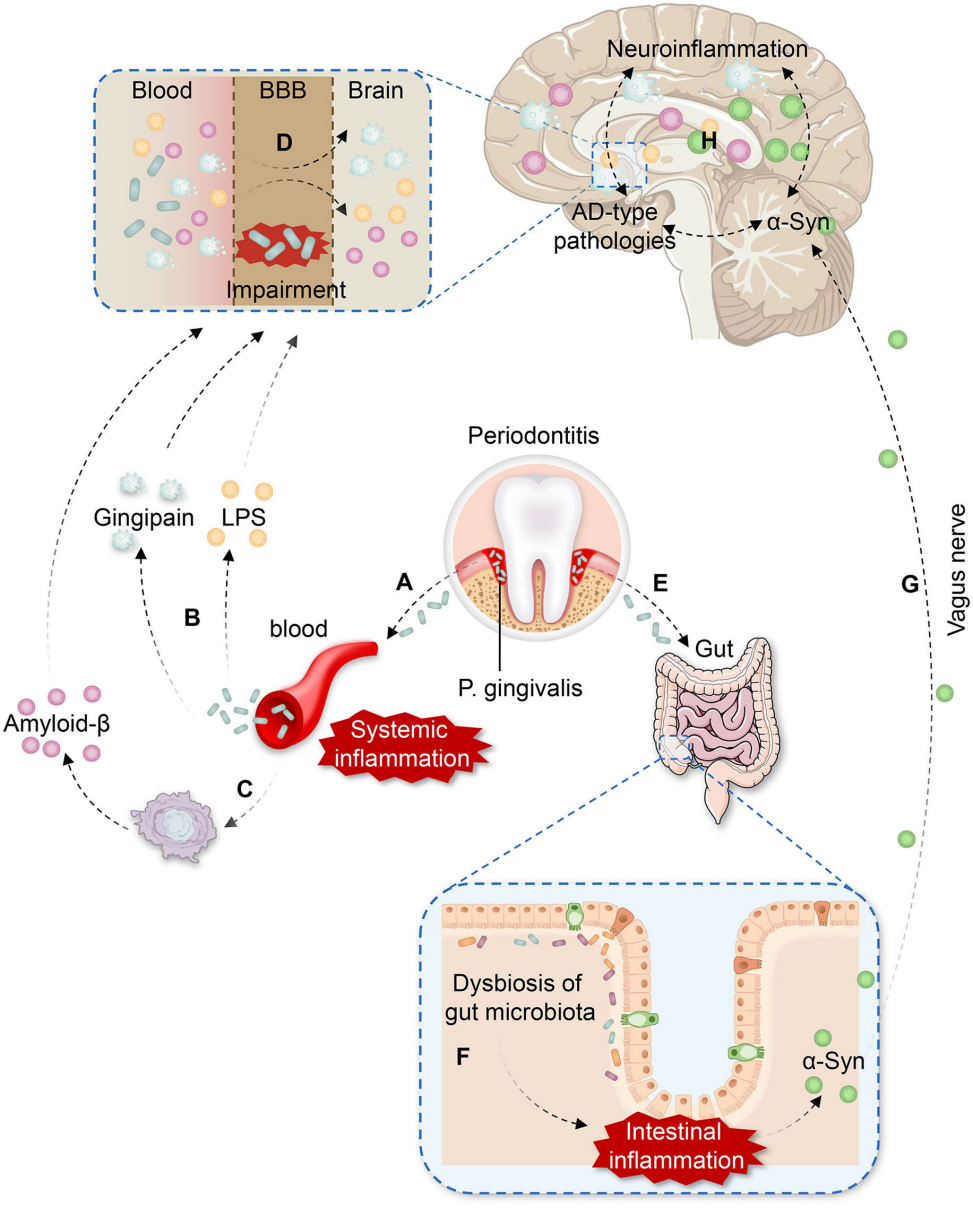
The potential pathways of *P. gingivalis* involvement in Parkinson's disease with cognitive impairment (PD-CI). **(A)** *P. gingivalis* enters the vasculature from the periodontal pocket, leading to bacteremia and systemic inflammation. **(B)** Gingipain and lipopolysaccharide (LPS) produced by *P. gingivalis* are present in the blood. **(C)** *P. gingivalis* infection induces the production of peripheral amyloid-β (Aβ) in inflammatory macrophages. **(D)** *P. gingivalis* impairs the function of the blood-brain barrier (BBB) by facilitating LPS, gingipain, and peripheral Aβ entry to the brain more easily, which causes neuroinflammation and Alzheimer's disease (AD)-type pathologies. **(E)** *P. gingivalis* is swallowed and enters the intestine. **(F)** *P. gingivalis* induces dysbiosis of the gut microbiota and intestinal inflammation, contributing to the accumulation of aberrant α-Synuclein (α-Syn) in the gut. **(G)** Aberrant α-Syn propagates from the gut to the brain *via* the vagus nerve. **(H)** Neuroinflammation, AD-type pathologies, and α-Syn promote each other effect in the brain, leading to a vicious cycle and resulting in PD-CI.

Based on the evidence mentioned above, we hypothesize that *P. gingivalis* may be involved in PD-CI through two pathways (Figure 1).

On the one hand, daily activities, such as tooth brushing and chewing, as well as dental procedures in patients with severe periodontitis can promote the entry of *P. gingivalis* into the vasculature from the periodontal pocket through ulcerated epithelium and lymphatic vessels (18, 28, 86). Additionally, *P. gingivalis* can destroy oral tissues to enter the bloodstream by gingipain (20). The entry of *P. gingivalis* into the bloodstream leads to bacteremia and the activation of the host immune response. Peripheral cytokines are subsequently increased (e.g., IL-1β, IL-2, IL-17A, and TNF-α), and systemic inflammation is induced, which facilitates the generation of peripheral Aβ. As *P. gingivalis* would impair the function of BBB, virulence factors, such as LPS, gingipain, and peripheral Aβ can easily enter the brain, resulting in the activation of microglia and astrocytes, causing neuroinflammation through TLRs. Ultimately neuroinflammation promotes aberrant α-Syn aggregation and AD-type pathologies in brain.

On the other hand, the oral-gut-brain axis may be another crucial mechanism. Patients with severe periodontitis can swallow large amounts of *P. gingivalis* each day. When entering the intestine, *P. gingivalis* may induce dysbiosis of gut microbiota, which simultaneously activate TLRs in the intestine, leading to intestinal inflammation and increased intestinal permeability. In addition to exacerbating systemic inflammation, the gut dysbiosis also contribute to the accumulation of aberrant α-Syn in the gut. Subsequently, α-Syn could propagate from the gut to the brain *via* the vagus nerve in a prion-like manner, aggravating α-Syn deposition and misfolding in the brain.

## Discussion

Collectively, the pathology of PD-CI has three major characteristics: convergence of α-Syn and Aβ pathologies, neuroinflammation, and dysbiosis of the gut microbiota. The currently available studies showed that *P. gingivalis* infection correlates with these features of PD-CI, underlining the association between *P. gingivalis* and PD, and between *P. gingivalis* and cognitive impairment, respectively. However, these studies have indirectly shown that *P. gingivalis* is associated with PD-CI, while no study has provided direct evidence of the association between *P. gingivalis* and PD-CI. Thus, the correlation between *P. gingivalis* and PD-CI needs further confirmation in subsequent studies.

## Author contributions

XZ conceived the concept of the review. DL did initial systemic search to find articles and drafted the manuscript with TR. XZ, HL, and GL modified the article. All authors contributed to the final manuscript and approved the submitted version.

## Funding

This study was supported by the Special Fund Project for Science and Technology Innovation Strategy of Guangdong Province and the High-level Hospital Construction Research Project of Maoming People's Hospital. XZ was supported by the Doctoral Research Foundation of Maoming People's Hospital.

## Conflict of interest

The authors declare that the research was conducted in the absence of any commercial or financial relationships that could be construed as a potential conflict of interest.

## Publisher's note

All claims expressed in this article are solely those of the authors and do not necessarily represent those of their affiliated organizations, or those of the publisher, the editors and the reviewers. Any product that may be evaluated in this article, or claim that may be made by its manufacturer, is not guaranteed or endorsed by the publisher.
